# Subclinical Congestion Evaluated by Point of Care Ultrasound (POCUS) at Discharge Predicts Readmission in Patients with Acute Heart Failure: Prognostic Cohort Study 

**DOI:** 10.24908/pocus.v9i2.17709

**Published:** 2024-11-15

**Authors:** Sergio Velasco Malagón, Estivalis Acosta­-Gutiérrez, José Atilio Nuñez­ramos, Sebastián Salinas, Guillermo Mora Pabón

**Affiliations:** 1 Universidad Nacional de Colombia Bogotá COL; 2 Department of Internal Medicine, Clínica Nueva El Lago Bogotá COL; 3 Department of Internal Medicine, Hospital Universitario Santa Clara Bogotá COL; 4 Instituto de Investigaciones Clínicas, Universidad Nacional de Colombia Bogotá COL; 5 Health Science Division, Hospital Universidad del Norte Barranquilla COL; 6 Emergency department, Hospital Universidad del Norte Bogotá COL; 7 Unidad de Cardiología, Hospital Universitario Nacional de Colombia Bogotá COL

**Keywords:** Congestive Heart Failure, VExUS, Volume Overload, Rehospitalization

## Abstract

**Background:** Heart failure (HF) is a complex entity that increases the risk of adverse outcomes. Point of care ultrasound (POCUS) allows easy lung and systemic venous congestion identification. Using ultrasound to detect sub-clinical congestion at discharge may help predict readmissions and mortality.** Outcomes:** The primary outcome was to address 30-day rehospitalization, and as a secondary outcome we investigated readmission and mortality in patients with residual congestion assessed with POCUS.** Methods:** A prospective prognostic cohort study was conducted at a tertiary-level institution in Colombia. Patients with acute decompensated heart failure (ADHF) at discharge were evaluated using POCUS through lung ultrasound (LUS), portal vein pulsatility (PVP), and a composite assessment of residual congestion. Inclusion criteria were ADHF, over 18 years old, with a “warm-wet” clinical profile. POCUS was performed using an ultraportable device using LUS and PVP. Statistical analysis used logistic regression models to estimate the association between ultrasound congestion and outcomes.** Results: **A total of100 patients were included. The population was mostly female, with a median age of 78 years; 59% were hypertensive, and 39% had type 2 diabetes. Median NT-ProBNP was 3878 pg/ml. At discharge, 55% of patient had an inferior vena cava (IVC) over 2 cm, 54% had interstitial syndrome, and 41% had PVP >30%. Regarding 30-day readmission, we found an odds ratio (OR) 7.22 (95% CI 2.7-19.3) for interstitial syndrome; for PVP >30%, an OR 24.61 (95% CI 7.7-78.1) and an OR 13.19 (95% CI 2.7-62.6) for composite of residual congestion. **Conclusion:** Patients with ADHF and sub-clinical congestion, evidenced in LUS and PVP, were more likely to have readmission within 30 days of discharge. These findings should be confirmed with clinical trials to assess the effectiveness of a POCUS-guided treatment.

## Background

Heart failure (HF) is a complex clinical syndrome characterized by ventricular dysfunction, leading to pulmonary and/or systemic congestion 1. The presence of signs and symptoms of congestion are the main feature in most patients with acute decompensated heart failure (ADHF) 2

Even though resolving congestion is the primary goal of treatment, this is not completely achieved during hospital admissions in up to 50% of patients. Rubio et al. showed that the presence of residual congestion increases the risk of readmission and mortality 3.

Guiding decongestive therapy based on signs and symptoms is neither sensitive nor specific 4. Rivas et al. found that approximately 40% of patients have residual congestion only identifiable with lung ultrasound (LUS) or abdominal vascular ultrasound 5. Beaubien-Soligny et al. published their experience using an ultrasound-based score to assess the systemic venous congestion, finding utility to predict acute kidney injury in post-cardiac surgery patients in the intensive care unit 6. However, the skill required to acquire some images, specifically hepatic vein and intra-renal Doppler, has halted the widespread use of this tool 7. Moreover, these ultrasound variables have not been widely evaluated in clinical settings outside critical care, and their clinical impact is unknown. It has been shown that the residual congestion using LUS adds prognostic information in patients with ADHF 8. Similarly, Bouabdallaoui et al. found that abnormal portal vein pulsatility (PVP) at the time of discharge in patients hospitalized for ADHF was associated with a higher risk of mortality 9. However, at this moment, there is little evidence about the prognostic value of residual congestion at discharge of general hospitalization after an episode of ADHF integrating abdominal and LUS.

Thus, we proposed to evaluate a cohort of patients treated for ADHF and assess the risk of rehospitalization according to systemic (organic congestion) and pulmonary congestion (alveolar interstitial syndrome). We hypothesized that patients discharged with residual congestion are more likely to die or be readmitted in 30 days than their peers without it. 

## Methods

This was a prospective prognostic cohort study conducted in a tertiary-level institution in Bogotá, Colombia. The ethics committee authorized the research protocol (Act No. 001 of 2022). The protocol was compliant with Helsinki’s Declaration. Every patient signed an informed consent prior to inclusion. 

Population

Patients with ADHF receiving in-hospital treatment with diuretics were eligible for the study. Data was obtained from clinical records from a convenience sample from July 2022 to May 2023.

Inclusion criteria were: 1) age over 18 years; 2) diagnosis of ADHF regardless of its etiology based on clinical history, images (chest x-ray, chest computed tomography (CT)-scan, echocardiography), and natriuretic peptides; and clinical profile with congestion without hypoperfusion (“warm-wet” Stevenson’s profile; 3) patients managed by an internal medicine physician during hospitalization according to clinical guidelines using intravenous furosemide for decongestion and presented no clinical signs of congestion (resolved shortness of breath, ankle edema, and rales 1.

Exclusion criteria included any pathology leading to errors in portal vein evaluation (cirrhosis, limited ultrasound window, abdominal masses, and hepatic surgery) and any history of interstitial lung disease, chronic pulmonary obstructive disease and pneumonia that could lead to the visualization of B-lines for other reasons, not being pulmonary congestion. 

Ultrasound assessment

All POCUS evaluations were performed by one researcher, internal medicine specialist (S.V.), and POCUS expert with more than five years of experience who was not involved in clinical care or discharge decisions. POCUS images were acquired with an ultra-portable ultrasound device (Butterfly IQ+. Guilford, CT) and a compatible electronic device (iPad Pro, Apple. Cupertino, California). Once patients were considered clinically decongested and deemed for discharge, a POCUS examination was performed.

POCUS was intended to evaluate pulmonary and systemic congestion. Pulmonary congestion was assessed with LUS seeking for B-lines, and systemic congestion was evaluated using portal vein flow and PVP. As well, a composite exposition of residual congestion was defined as the presence of any echographic variable suggestive of overload (IVC >2 cm, PVP above 30%, B-pattern in LUS or pleural effusion).

Lung images were obtained using the “Lung” preset and according to the BLUE protocol previously reported 10. The B-pattern or interstitial syndrome was diagnosed when more than two B-lines were observed in two areas bilaterally. Pleural effusion was evaluated in BLUE protocol zone 3. 

**Figure 1:**
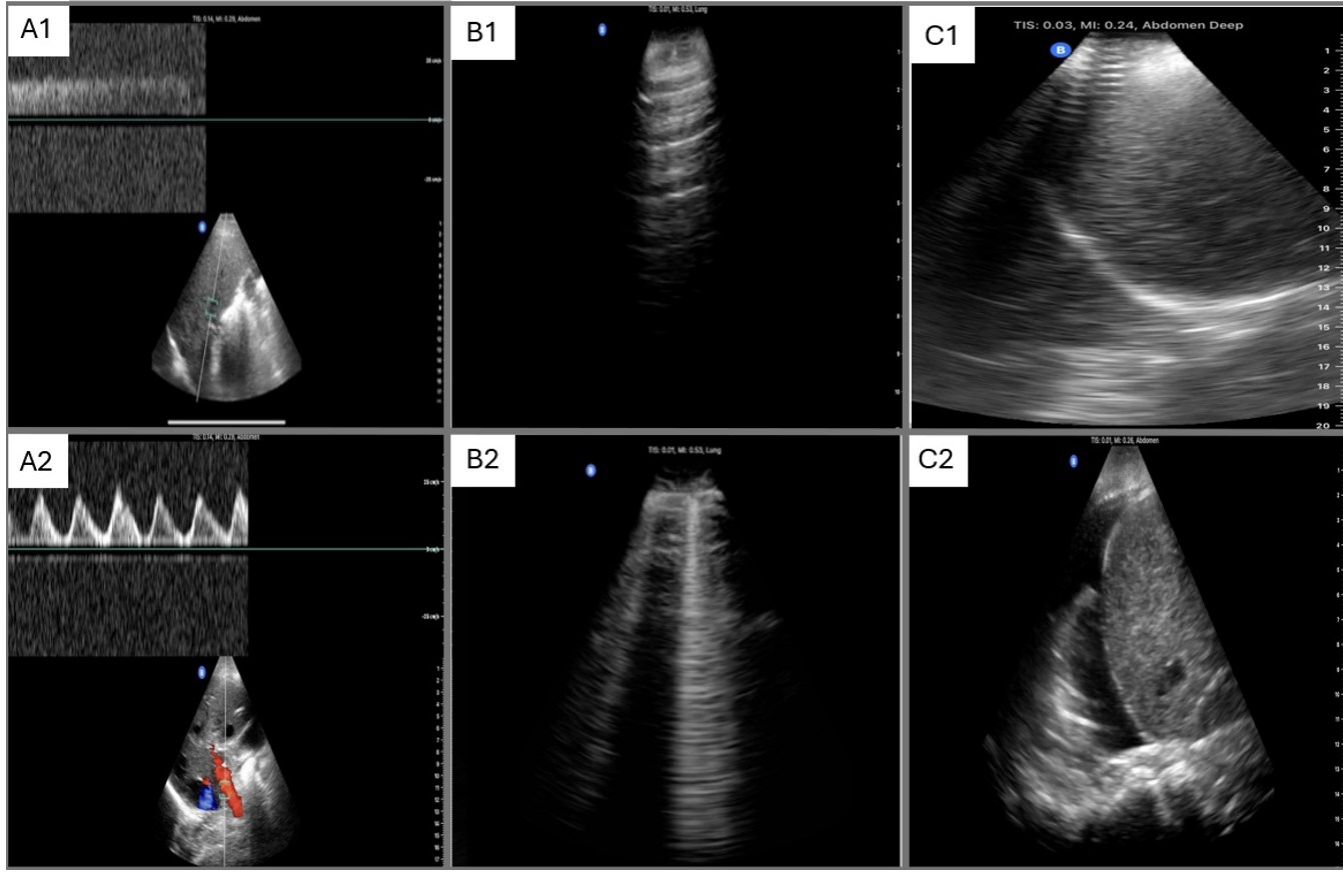
Point of care ultrasound (POCUS) evaluation of congestion.A. Portal vein pulsatility evaluated in the hepatic window with a pulsed wave Doppler. A1. Portal vein pulsatility less than 30%. A2. Portal vein pulsatility above 30%. B. Lung ultrasound. B1. A-lines in a lung without congestion. B2. B-lines in the context of Alveolar interstitial syndrome. C. Lung ultrasound for the evaluation of pleural effusion. C1. No pleural effusion. C2. Pleural effusion in the right lung.

Using the “Abdominal” preset, the IVC was assessed at end-expiration. The diameter was measured 2 cm away from the hepatic vein entrance. Images and measures were performed in long and short axes to avoid errors. The mean value of two measures was reported. The portal vein was assessed at end-expiration, with no more than 30° for pulsed-wave Doppler interrogation. The pulsatility index was considered positive when its value was equal to or above 30% pulsatility. Measures and techniques for portal vein evaluation were the same as those used for the VExUS score protocol (Figure 1) 6. 

**Table 1:** Characterization of clinical, sociodemographic, and outcome variables regarding all patients and comparison between congestive and non-congestive patients. The statistical test for quantitative variables was the Wilcoxon test. Fisher’s test was used for categorical variables.

**Characteristics of patients according to whether they present any point of care ultrasound (POCUS) findings of congestion **				
	**All** **n=100**	**Congestive** **n=74**	**Non-congestive** **n=26**	**P** **value**
Age - median (IQR)	78 ±14	77±13	83±14	0.29
Female- % (CI)	55 (45.24-64.38)	74.54 (61.81-84.18)	14 25.45(15.81-38.3)	1
**POCUS** **Evaluation** - % (CI)IVC > 2 Portal vein pulsatility Pattern B Pleural effusion	55 (45.24-64.38) 41 (31.86-50.79) 54 (44.26-63.43) 34 (24.56-42.69)	74.32(63.34-82.90)55 .04(44.09-66.18)72.97(61.90-81.76)43 (33.81-55.90)	0(0-4.93)	-
Weight at first meet- median (IQR)	62 ±19	64±20.75	60.5 ±17.75	0.39
Weight at discharge- median (IQR)	58 ±19	58.50±19.5	56.50±17	0.89
Intensive care unit stay - % (CI)	14 (8.52-22.13)	85.71(60.05-95.99)	14.28(4-39.94)	0.346
Chronic Kidney Disease - % (CI)	29 (21.01-38.53)	93.1(78.03-98.08)	6.89 (1.91-21.96)	0.005*
Acute Kidney Injury - % (CI)	45 (35.61-54.75)	86.66(73.83-93.74)	13.33(6.25-26.27)	0.012*
Dialysis - % (IC)	6 (2.77-12.47)	100 (60.96-100)	0(0-39.03)	0.33
LVEF - median (IQR)	55 ±25	55±20	59±18.5	0.0086*
NT-ProBNP - median (IQR)	3878	5766±9778	1464±333	0.000*
Aortic insufficiency - % (CI)	11 (6.25-18.63)	81.81 (52.3-94.86)	18.18(5.13-47.69)	0.72
Mitral regurgitation - % (CI)	41 (31.86-50.79)	87.80(74.45-94.67)	12.19 (5.32-25.54)	0.011*
Tricuspid regurgitation - % (CI)	36 (27.27-45.76)	88.88(74.68-95.59)	11.11(4.4-25.31)	0.016*
Aortic stenosis - % (CI)	4 (1.56-9.83)	75(30.06-95.44)	25 (4.55-69.93)	1
Mitral stenosis - % (CI)	3(1.02-8.45)	66.66(20.73-93.85)	33.33(6.14-79.23)	1
Rehospitalization at 30 days -% (CI)	41 (31.86-50.79)	95.12(83.86-98.65)	4.87 (1.34-16.13)	0.000*
Death at 30 days - % (CI)	7 (3.43-13.74)	100 (64.56-100)	0 (0-35.43)	0.18

Images and measures were stored and de-identified. A second researcher (J.N.), a POCUS expert, blindly evaluated the images and reported diagnoses with every case (pulsatile portal vein yes/no). If no agreement was reached in a specific image, a third researcher (S.S.) was consulted. Inter-observer agreement was calculated.

Physicians in charge of clinical care did not have access to ultrasound evaluation. The researcher performing the POCUS evaluation did not have access to clinical records before the POCUS exam. 

Outcomes

Patients had a telephone interview to assess readmission and mortality status. The primary outcome was a 30-day readmission due to ADHF. The secondary outcome was 30-day mortality. Acute kidney injury was assessed during hospitalization. 

Statistical Analysis

The sample size was obtained based on Peduzzi considerations^8^ on the optimal number of events per variable in a logistic regression model, where at least ten events were necessary to diminish the risk of bias in estimating the model. Therefore, we included four variables in our model for 40 events.

Univariate analysis reported measures of central tendency and data dispersion for continuous variables according to data distribution. Categorical variables are expressed in absolute and relative frequencies. Comparisons between congestive and non-congestive patients were made using the Wilcoxon rank sum test, and since many of the continuous variables did not follow a normal distribution, the association between categorical variables was performed using chi-square and Fisher’s tests. Multivariate analysis was performed through a logistic regression model assessing the risk of 30-day readmission. The model included demographic variables, known covariates, pulmonary ultrasound congestion in LUS, systemic ultrasound congestion defined as PVP, and a composite exposition of residual congestion that included any of the following: pleural effusion, increased portal pulsatility, IVC >2 cm without inspiratory collapse and/or pulmonary B profile. Multicollinearity, outliers, and goodness-of-fit were evaluated in each regression model. Results are expressed as odds ratios (OR) with a 95% confidence interval (CI). Detailed information on statistical analysis is in the supplementary data. Additionally, an inter-rater agreement was explored using the Kappa coefficient.

## Results

During the time of the study, a total of 100 patients with ADHF had a POCUS evaluation before discharge. The population was predominantly female, with a median age of 78 (IQR±14) years old, 59% hypertensive, and 39% had a history of type 2 diabetes. Most patients had decompensation of chronic HF. Among all patients, 29% had chronic kidney disease, and 6% were on renal replacement therapy. The median NT-ProBNP was 3878 pg/ml. Patient characteristics are reported in Table 1.

Echocardiography during hospitalization showed a left ventricle ejection fraction (LVEF) mean of 48%; 36% had tricuspid regurgitation, 41% mitral regurgitation, and 4% aortic stenosis. Of all participants, 29 patients had HF with reduced ejection fraction (HFrEF). 

Regarding POCUS findings at discharge, 54% had interstitial syndrome, 41% presented a portal vein with abnormal pulsatility, and 71% the composited exposition of residual congestion. 

Inter-rater agreement was assessed using the Kappa coefficient. Analysis showed a 0.85 agreement with a 0.09 standard error.

**Figure 2:**
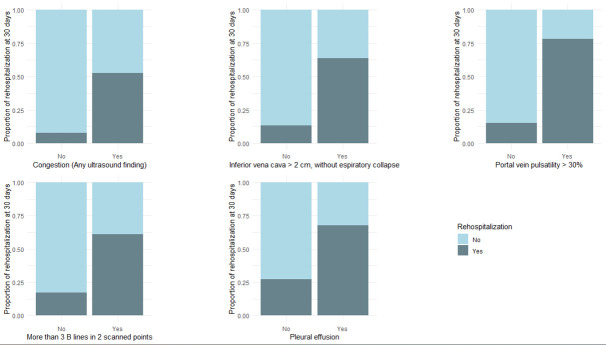
A stacked bar chart of the proportions of patients with and without readmission according to the evidence of residual congestion with point of care ultrasound (POCUS) evaluation.A. Proportion of rehospitalization of patients according to their findings of congestion (any ultrasound finding). B. Proportion of rehospitalization of patients according to their findings of congestion using inferior vena cava. C. Proportion of rehospitalization of patients according to portal vein pulsatility. D. Proportion of rehospitalization of patients according to the presence of B-lines in lung ultrasound. E. Proportion of rehospitalization of patients according to the presence of pleural effusion in lung ultrasound.

In bivariate analysis, abnormal PVP >30%, IVC diameter >2 cm, and interstitial syndrome in LUS were more frequent in residual congestive patients (Figure 2). Moreover, these variables were associated with the incidence of acute kidney injury and readmission with p-values under 0.05 (see supplementary data).

Patients with residual congestion at discharge had a median weight loss of 4 kg, elevated Pro-BNP levels, reduced LVEF, and a higher incidence of intensive care unit admissions and acute kidney injury. Readmissions were more frequent in residual congestive individuals, and all-cause mortality events were presented mainly in this group, as is shown in Figure 2. 

According to logistic regression models evaluating factors associated with 30-day readmission, we found an OR 7.22 (95% CI 2.7-19.3) for interstitial syndrome in LUS. Seemingly, PVP >30% had an OR 24.61 (95% CI 7.7-78.1), and the composite of residual congestion had an OR 13.19 (95% CI 2.7-62.6) (Table 2).

Treatment according ejection fraction is detailed in Table 3. A detailed report on each logistic regression model and additional analyses is included in the supplementary data. 

**Table 2:** The odds ratio of each independent variable in association with 30-day readmission for point of care ultrasound (POCUS) variables, estimated with logistic regression models #1, #2 and #3, respectively.

**Variable**	**OR** adjusted*	**95%CI**
Interstitial lung syndrome	7.22	2.7 - 19.3
Portal vein pulsatility >30%	24.61	7.7 - 78.1
Residual congestion**	13.19	2.7 - 62.6

*Adjusted OR corresponds to models 1, 2, and 3. See supplementary data. ** Any of the following: pleural effusion, increased portal pulsatility, >2 cm inferior vena cava, interstitial syndrome ** Any of the following: pleural effusion, increased portal pulsatility, >2 cm inferior vena cava, interstitial syndrome

**Table 3:** Treatment of heart failure according to Left Ventricular Ejection Fraction

	Reduced LVEF	Preserved LVEF
ACEI	0.37 (IC 95% 0.22-0.56)	0.42 (IC 95% 0.31-0.54)
ARNI	0.51 (IC 95% 0.33-0.69)	0.0
ACEI or ARNI	0.86 (IC 95% 0.68-0.94)	0.42 (IC95 0.31-0.54)
SGLT2i	0.93 (IC 95% 0.75-0.98)	0.32 (IC 95% 0.22-0.44)
MRA	0.93 (IC 95% 0.75-0.98)	0.21 (IC 95% 0.13 - 0.32)
Beta-blocker	1	0.57 (IC 95% 0.45 - 0.68)

ACEI: Angiotensin-converting enzyme inhibitors, ARNI: Angiotensin Receptor–Neprilysin Inhibitor, SGLT2i: Sodium-glucose co-transporter-2 inhibitors, MRA: mineralocorticoid receptor antagonist.

## Discussion

In this prospective study of ADHF, we found that signs of residual congestion at discharge were associated with hospital readmission and all-cause mortality at 30 days. This study is novel as our population had no evidence of congestion upon physical exam; however, the presence of interstitial syndrome and PVP were still associated with higher risk of readmission and mortality.

These findings show that discharging HF patients without a POCUS evaluation to comprehensively assess left-sided and right-sided congestion is detrimental for this group of patients. Although we did not pursue mortality analysis because of the low number of outcomes, we found that all patients deceased at 30 days had residual congestion at discharge. LUS has a long history of accurate, rapid, and reliable evaluation of pulmonary congestion 11. B-lines and interstitial syndrome are clearly related to pulmonary edema, persistent congestion, and pulmonary-capillary wedge pressure 12. IVC dilation is strongly related to systemic venous congestion and is the first step to performing the VExUS score. PVP has been described in patients with high filling pressures and has an impact in predicting pressure driven end organ damage 13, 14.

Our study relates to the work performed before by Rattarasarn and Gargani 15, 16. They showed a higher risk of readmission after finding over 12 B-lines and over 15 B-lines in LUS. Drawing from the POCUS idea, we proposed a rapid and simple LUS evaluation according to BLUE protocol. Three B-lines in two BLUE points bilaterally defined interstitial syndrome. We are sure that simplifying POCUS evaluation without diminishing accuracy and prediction is the best way to stimulate the everyday use of POCUS in real clinical practice. Our data confirms that discharging patients with persistent B-lines is not safe, and nearly assures that the patient will be readmitted. 

Regarding right-sided venous systemic congestion, published papers report the high specificity and predictive value of the VExUS score for readmission, all-cause mortality, and acute kidney injury. In research done by Torres-Arese et al.^17^ with a population comparable to our patients, the VExUS score was performed at admission, discharge, and 90-day follow-up. They analyzed all VExUS score variables and found that the most congested state (VExUS 3), portal pulsatility >50%, and monophasic intra-renal flow pattern were associated with in-hospital mortality. Seemingly, a dilated >2 cm IVC and a monophasic intra-renal flow pattern were associated with readmission at a 90-day follow-up visit. We analyzed only two variables of the VExUS score and analyzed specifically PVP. Considering the high correlation between altered flow in this vessel and tissue congestion, we could demonstrate that evaluating only this Doppler pattern is accurate in predicting readmissions. 

The research published by Dr. Pugliese et al.^18^ evaluated the IVC, LUS, and intra-renal flow. They demonstrated the association of these variables with all-cause mortality or readmission with a hazard ratio of 26.7 (95%CI 12.4-63.6) – a value close to our results with PVP (OR 24.61 - 95% CI 7.7-78.1). Dr. Pugliese did not evaluate PVP; only intra-renal flow was evaluated. This approach demands robust ultrasound equipment (not ultra-portable such as those used in POCUS), highly trained personnel, and possibly frequently missed evaluations due to difficulties identifying the proper site to perform the Doppler assessment. This late statement about the difficulty and expert training is affirmed by VExUS authors 7.

Residual congestion with any of the following – pleural effusion, increased portal pulsatility, >2 cm IVC, or interstitial syndrome – addressed by POCUS was associated with readmission (OR:13.19). This data shows that attempts to correct these abnormalities should be encouraged in patients with HF and might have an impact in readmission. We emphasize that isolated parameters should not be taken as a unique trigger for changing treatment.

We also found a strong correlation between PVP >30% and acute kidney injury (X^2^: 22 P< 0.005, supplementary data) suggesting the impact of congestive nephropathy in this outcome.

These findings are consistent with previously published data by Rihl^19^ and Argaiz^20^ showing the importance of assessing congestion in every patient with acute kidney injury.

Treatment was established according to the ejection fraction per current HF guidelines 4. In HErEF, adequate treatment was present above 85% (Table 3). In patients with HFpEF, the use of inhibitors of sodium-glucose co-transporters was below 50%. The evidence showing the impact in this scenario is recent, so we expect it to present higher with time.

LUS and PVP measurements can be performed at the bedside quickly and reliably with an ultra-portable ultrasound device. We intentionally did not evaluate hepatic veins, considering the need for electrocardiogram tracing to accurately determine systolic and diastolic waves at this level. Moreover, in daily clinical practice, performing all VExUS variables and interpreting each one is difficult and time-consuming. 

The present study has several limitations. It is a single-center cohort study that included a relatively small proportion of patients with HF, which may lead to limited generalizability. It is important to consider the scenarios in which the use of LUS, IVC, and PVP may not be interpretable. The POCUS evaluations performed by one expert may affect the application of these results, although we managed this bias using a second interpretation of images and calculating the Kappa agreement coefficient. We did not perform a mortality analysis considering statistical issues due to sample size; however, we did a thorough regression analysis, and the assumptions of the model were fully met (supplementary data).

The strengths of our study include the fact that real practice is easily obtainable and that POCUS variables are highly reproducible (IVC, LUS, PVP). This simple and accurate approach allows applicability in clinical scenarios using a portable device. We could establish a well-grounded cohort of patients with a well-established diagnosis and a close follow-up within an adequate period (30 days). Our statistical analysis was strong, using well-constructed models and addressing possible biases by controlling with logistic regression. 

Based on the results of this study and acknowledging the risk of readmission and mortality of congestive patients, future research is warranted. This should evaluate the impact of decongestive therapy guided by POCUS findings before hospital discharge. 

## Conclusion

In our study, patients with an ADHF episode and sub-clinical congestion evidenced in LUS (interstitial syndrome) and PVP (>30%) were more likely to have a readmission within 30 days of discharge. This simplified evaluation permits its use by every clinician treating acute HF. These findings should be confirmed with clinical trials to assess the effectiveness of a POCUS-guided treatment and pre-discharge evaluation.

## Statement of ethics

The ethics committee authorized the research protocol (Act No. 001 of 2022). The protocol was compliant with Helsinki’s Declaration. Every patient signed an informed consent prior to inclusion.

## Disclosure Statement 

The authors declare that they have no competing interests.
